# Associations between Antibodies to a Panel of *Plasmodium falciparum* Specific Antigens and Response to Sub-Optimal Antimalarial Therapy in Kampala, Uganda

**DOI:** 10.1371/journal.pone.0052571

**Published:** 2012-12-19

**Authors:** Chris E. Keh, Aashish R. Jha, Bridget Nzarubara, David E. Lanar, Sheetij Dutta, Michael Theisen, Philip J. Rosenthal, Grant Dorsey, Douglas F. Nixon, Bryan Greenhouse

**Affiliations:** 1 Department of Medicine, University of California San Francisco, San Francisco, California, United States of America; 2 Infectious Diseases Research Collaboration, Kampala, Uganda; 3 Division of Malaria Vaccine Development, Walter Reed Army Institute of Research, Silver Spring, Maryland, United States of America; 4 Department of Clinical Biochemistry and Immunology, Statens Serum Institut, Copenhagen, Denmark; Kenya Medical Research Institute (KEMRI), Kenya

## Abstract

**Background:**

Antibodies are important in the control of blood stage *Plasmodium falciparum* infection. It is unclear which antibody responses are responsible for, or even associated with protection, partly due to confounding by heterogeneous exposure. Assessment of response to partially effective antimalarial therapy, which requires the host to assist in clearing parasites, offers an opportunity to measure protection independent of exposure.

**Methods:**

A cohort of children aged 1–10 years in Kampala, Uganda were treated with amodiaquine+sulfadoxine-pyrimethamine for uncomplicated malaria. Serum samples from the time of malaria diagnosis and 14 days later were analyzed for total IgG to 8 *P. falciparum* antigens using a quantitative indirect ELISA. Associations between antibody levels and risk of treatment failure were estimated using Cox proportional hazard regression.

**Results:**

Higher levels of antibodies to apical membrane antigen 1 (AMA-1), but to none of the other 7 antigens were significantly associated with protection against treatment failure (HR 0.57 per 10-fold increase in antibody level, CI 0.41–0.79, p = 0.001). Protection increased consistently across the entire range of antibody levels.

**Conclusions:**

Measurement of antibody levels to AMA-1 at the time of malaria may offer a quantitative biomarker of blood stage immunity to *P. falciparum*, a tool which is currently lacking.

## Introduction

Malaria remains one of the leading causes of morbidity and mortality in children living in sub-Saharan Africa [1]. The development of acquired immunity to *Plasmodium falciparum* prevents much of this morbidity in older children and adults, but it is slow to develop and requires repeated episodes of malaria. It has been shown that naturally acquired antibodies to *P. falciparum* can control malarial parasitemia [2], [3], yet which antibody responses lead to protection remains unknown. Antibodies directed against a number of *P. falciparum* proteins have been associated with a lower risk of malaria [4]–[6]. However, it is difficult in such studies to distinguish decreased risk due to immunologic protection from decreased malaria incidence due to a lack of parasite exposure [7]–[9], making it challenging to identify associations between antibody responses and the incidence of malaria. Indeed, partly due to this challenge, we lack widely accepted biomarkers of antimalarial immunity.

Assessing the response to partially effective antimalarial therapy offers an opportunity to estimate the level of blood stage antimalarial immunity independent of knowledge of prior exposure. In this context, acquired immunity enhances the efficacy of antimalarial therapy such that increasing immunity affords increasing ability of sub-optimal therapy to eliminate parasitemia [10], [11]. Drug efficacy studies of partially effective antimalarial regimens therefore offer an opportunity to assess associations between antibody responses and clinically relevant antimalarial immunity.

We have previously described an association between clinical surrogates of host immunity and protection from failure after treatment with amodiaquine plus sulfadoxine-pyrimethamine (AQ+SP) in a cohort of children in Kampala, Uganda [12]. To determine whether antibody responses to specific *P. falciparum* antigens were associated with clearance of parasitemia, we measured IgG responses to 8 parasite antigens previously associated with clinical protection from malaria [6], [13]–[16] and analyzed associations between these responses and treatment outcomes.

## Materials and Methods

### Study Site and Participants

The clinical study was conducted in Kampala, Uganda between November 2004 and December 2008 and has been previously described [17], [18]. Briefly, children from 1–10 years of age were randomly selected from the Mulago III parish in Kampala and enrolled in a randomized trial of combination antimalarial therapies. Caretakers of study participants were asked to bring their children to the clinic for any febrile episode or illness. Uncomplicated malaria was defined as fever (tympanic **≥**38.0**°**C or history of fever in previous 24 hours), parasitemia detected by microscopy, and absence of complicated malaria defined by evidence of severe disease [19], inability to stand or drink, lethargy, recent convulsions, persistent vomiting, or parasite density ≥500,000/µl. The current study examines subjects that were randomized to receive AQ+SP for all episodes of uncomplicated malaria. Children received active follow-up for 28 days. Serum samples were collected at the time of diagnosis (Day 0) and 14 days following treatment (Day 14) and stored at −80°C. Recurrent episodes of malaria within 63 days of initial treatment were genotyped to distinguish new infection and recrudescence (treatment failure) using 6 loci [20]. Recurrent malaria that occurred >63 days after a prior episode was considered a new infection. Treatments of recrudescent infections (i.e. retreatments of treatment failures), non-falciparum malaria, early treatment failures [21], subjects who did not complete therapy, and those without genotyping results were excluded from the current analysis. Routine assessments for asymptomatic parasitemia occurred every 30 days.

### Antibody Testing by Enzyme-Linked Immunosorbent Assay (ELISA)

96-well microtiter plates (Immulon 4HBX, Thermo Scientific, USA) were coated overnight at 4°C with antigens of interest diluted in 0.01M phosphate buffered saline (PBS). All blocking and wash steps occurred at a volume of 200 µl/well. Plates were washed twice with a solution of PBS containing 0.05% Tween20 (PBST), followed by a 1-hour block with a solution containing 5% Blotto, non-fat dry milk (Santa Cruz Biotechnology, Santa Cruz, CA) in PBS (PBSB) at 37°C. Plates were again washed twice with PBST, followed by addition of 100 µl/well patient serum samples diluted to 1∶200 in PBSB. Patient samples were run in duplicate, along with negative controls (PBS or pooled serum from *P. falciparum* unexposed donors). Following a 2-hour incubation at 37°C, the plates were washed 3 times with PBST and 100 µl/well of alkaline-phosphatase-conjugated goat anti-human IgG (Jackson Immunoresearch, West Grove, PA) diluted to 1∶1000 in PBSB was added. Following a 1-hour incubation at 37°C, wells were extensively washed 6 times with PBST. BluePhos Microwell Phosphatase Substrate solution (100 µl/well, KPL, Gaithersburg, MD) was used to develop the plates, which were read at 650 nm on a Spectramax M2 ELISA plate reader using Softmax Pro Software v5.2 (Molecular Devices, Sunnyvale, CA). Following the addition of substrate, optical densities (OD) were collected every 4 minutes for a total of 40 minutes. Serial 3-fold dilutions (1∶33 to 1∶72,900) of pooled serum from 50 African adults living in Kampala were analyzed on every plate as a positive control and to produce a standard curve for relative antibody quantification. The plate showing the greatest dynamic range for the standard curve was used. OD values were converted to antibody level expressed in arbitrary units (AU), where an AU of 100 was equivalent to the antibody level in the pooled serum. Samples with OD values above the linear range of the standard curve were further diluted up to two times to a maximum dilution of 1∶10,000 and re-analyzed to obtain a better quantitative estimate of antibody level.

The following recombinant *P. falciparum* proteins were used for ELISA, with coating concentration indicated in parentheses: apical membrane antigen 1 (AMA-1) from the 3D7 strain expressed in *Escherichia coli* (0.1 µg/ml) [22]; C-terminal 19 kDa fragment of merozoite surface protein 1 (MSP-1) from the FVO strain expressed in *Saccharomyces cerevisiae* (0.25 µg/ml) [23]; merozoite surface protein 2 (MSP-2) from the 3D7 strain expressed in *E. coli* (0.25 µg/ml) [24]; merozoite surface protein 3 (MSP-3) polypeptide corresponding to the C-terminal region, produced in *E. coli* (1 µg/ml) [25]; the non-repetitive amino-terminal region of glutamine rich protein (GLURP-R0) from the F32 strain expressed in *E. coli* (0.5 µg/ml) [26]; and the carboxy-terminal repeat region of glutamine rich protein (GLURP-R2) from the F32 strain expressed in *E. coli* (0.25 µg/ml) [26].

In addition, the following synthetic peptides (15 µg/ml, Invitrogen, Grand Island, NY) were used in ELISA: the (NANP)_5_ repeat peptide for circumsporozoite protein (CSP); and the central amino acid repeat sequence LAKEKLQGQQSDLEQERLAKEKLQEQQSDLEQERLAKEKLQ for liver-stage antigen (LSA).

### Statistical Analysis

Antibody prevalence and fold change between Day 0 and Day 14 were calculated for descriptive statistics. To determine antibody prevalence, the positive cutoff was set at 3 standard deviations above the mean response in samples from twenty healthy adults who have never been diagnosed with malaria or traveled within malaria endemic regions (negative controls). Fold change in antibody levels between Day 0 and 14 was determined by the difference in log-transformed values during the course of malaria follow up in an individual. Given that a number of subjects had multiple episodes of malaria, repeated measures within the same individual were accounted for by using generalized estimating equations (GEE) to estimate antibody prevalence, geometric mean antibody level, and the fold change of antibody level between Day 0 and 14. Pairwise correlation coefficients were obtained to determine correlation of antibody responses between each other. For all subsequent analyses, continuous antibody levels were used after log-transformation, truncating low values at the mean response in negative controls.

To determine associations between epidemiologic factors associated with *P. falciparum* exposure and antibody levels on Days 0 and 14, we evaluated the following predictor variables: age at study enrollment (to allow for independent evaluation of the effects of age and calendar time) as a continuous variable; date of the malaria episode as a continuous variable; whether or not the subject lived within 50 meters (m) of a swamp, previously shown to be associated with higher malaria incidence and higher antibody responses [9], [27]; asymptomatic parasitemia (defined as the presence of a positive blood smear in the absence of fever, occurring at least 28 days after and 5 days before treatment for malaria) in the 180 days prior to malaria diagnosis [12]; and log parasite density at the time of malaria. Associations between predictor variables of interest and antibody levels were determined using multivariate linear regression, weighting by the inverse of the number of observations contributed by an individual to adjust estimates for repeated measures. Inference was obtained using 1000 bootstrap replicates.

To assess associations between antibody responses and the risk of treatment failure, hazard ratios were estimated for each response using Cox proportional hazard regression, adjusting for age at enrollment and parasite polymorphisms previously associated with treatment failure [12]. Robust inference accounting for repeated measurements in the same subject was performed using the grouped jackknife method. Assessing response to antimalarial treatment via survival analysis was performed because of censoring of treatment failure by new infections [28]. We tested the proportional hazards assumption using Schoenfeld residuals and found that this was not violated by any of the 8 antibody responses. Statistical analyses were performed using Stata (StataCorp, v11.2) and R (R Foundation for Statistical Computing, v2.13) [29] and a p value of <0.05 was considered significant.

### Ethical Statement

Written informed consent for their participation in the study was obtained from the children’s parents or guardians, in accordance with the Declaration of Helsinki. Approval was granted by the Uganda National Council of Science and Technology, the Makerere University Research and Ethics Committee, and the University of California, San Francisco Committee on Human Research.

## Results

### Characteristics of Patients and Samples

**Figure 1:**
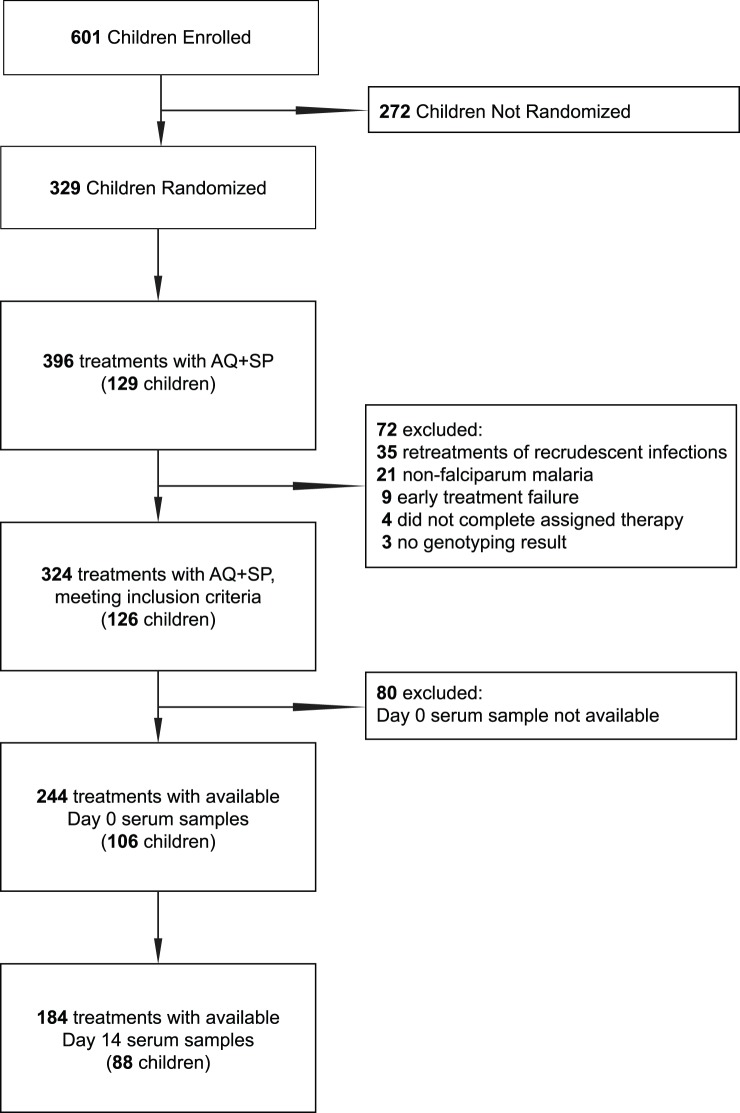
Sample inclusion. Malaria treatments with amodiaquine+sulfadoxine-pyrimethamine (AQ+SP) and available samples for inclusion in the study. Further details on children not randomized have been previously described [17].

Over the course of the study, 601 total subjects were enrolled and 329 children were randomized in the clinical trial of combination antimalarial therapy. Children randomized to AQ+SP were followed up to 28 months on this therapy. The prevalence of asymptomatic parasitemia in this cohort was 18.6% at enrollment and fell to 2.3% during the study [17]. 129 children were treated with AQ+SP for a total of 396 treatments. The 63-day risk of recrudescence (treatment failure) in this treatment arm was 11% [12]. Samples from 106 participants meeting inclusion criteria were available for antibody analysis, representing 244 treatments (Day 0) and 184 follow-up samples (Day 14) (Figure 1). In this subset, 29 of the 244 treatments resulted in treatment failure, giving a 63-day risk of treatment failure of 13%; there was a median of 2 treatments per subject (range 1–11); the mean age at enrollment was 6.0 years (range 1.2–10.0 years); 45.9% of the participants lived within 50 meters of a swamp that bordered the study site; and asymptomatic parasitemia was detected within 180 days prior to malaria diagnosis for 29.0% of the treatments.

### Antibody Responses at the Time of Malaria Diagnosis and 14 Days Later

The prevalence of antibodies directed against LSA, AMA-1, MSP-1, and MSP-2 at the time of malaria diagnosis was greater than 50% (Table 1). Antibody responses to each of the 8 antigens tested were significantly correlated (range: 0.2–0.7, p<0.001 for all pair-wise comparisons). Despite the overall high prevalence, geometric mean antibody levels at the time of diagnosis were at least 10-fold lower than levels seen in malaria exposed adults, though with a wide range across individuals. The level of antibodies directed against all antigens except CSP increased significantly in the 14 days following diagnosis and treatment, with the greatest increases seen in responses to the three merozoite surface proteins (2.2–3.1 fold increase, p≤0.001). Thus, in this cohort, antibody responses to multiple antigens were frequently detected at the time of malaria but at varying levels, and these responses were boosted in the 14 days following malaria diagnosis and treatment.

**Table 1:** Antibody responses to *Plasmodium falciparum* antigens.

Antigen	Antibody Prevalence^a^, % (95% CI)		Antibody Level, AU^b^, Geometric Mean, (Range)		Fold increase between Day 0 and 14 Levels
	Day 0	Day 14	Day 0	Day 14	(95% CI)
CSP	37.8 (30.3–46.0)	39.0 (30.4–48.3)	10.4 (1.2–3485.9)	10.0 (1.2–2901.8)	1.0 (0.9–1.2), p = 0.5
LSA	87.4 (81.8–91.4)	93.1 (87.4–96.4)	6.4 (0.06–10000.0)	10.5 (0.06–4265.9)	1.6 (1.3–2.1), p≤0.001
AMA-1	62.6 (53.5–70.9)	73.6 (64.8–80.8)	1.9 (0.05–2360.4)	3.3 (0.05–1899.3)	1.5 (1.0–2.1), p = 0.03
MSP-1	94.8 (90.6–97.1)	99.5 (96.2–99.9)	6.3 (0.02–3597.0)	17.0 (0.06–1154.8)	2.6 (1.9–3.6), p≤0.001
MSP-2	86.5 (79.6–91.3)	96.8 (93.1–98.5)	2.0 (0.01–589.6)	6.5 (0.01–1072.3)	3.1 (2.3–4.2), p≤0.001
MSP-3	15.2 (10.4–21.6)	34.6 (26.9–43.3)	3.3 (0.8–1323.5)	7.0 (0.8–1051.3)	2.2 (1.7–2.8), p≤0.001
GLURP-R0	25.5 (19.3–33.0)	31.1 (23.2–40.3)	3.5 (0.6–208.6)	4.1 (0.6–126.3)	1.2 (1.0–1.5), p = 0.03
GLURP-R2	40.4 (32.5–48.9)	52.8 (43.4–62.0)	7.1 (0.6–2216.3)	10.0 (0.6–843.5)	1.4 (1.1–1.8), p = 0.01

a Positive cutoff = mean of 20 *P. falciparum* unexposed controls +3 standard deviations.

b AU = arbitrary units, where an AU of 100 is equivalent to the antibody level of pooled serum from 50 African adults.

Note: Generalized estimating equations (GEE) were used to estimate antibody prevalence, geometric mean antibody level, and fold change.

Circumsporozoite protein (CSP); liver stage-antigen (LSA); apical membrane antigen 1 (AMA-1); merozoite surface protein 1, 2, or 3 (MSP-1, MSP-2, MSP-3); amino- or carboxy-terminal region of glutamine rich protein (GLURP-R0, GLURP-R2).

### Associations between Factors Indicative of *P. falciparum* Exposure and Antibody Levels at the Time of Malaria Diagnosis

Antibody responses to AMA-1 at the time of diagnosis and treatment were most strongly associated with age at enrollment (44% increase per year of age) and living within 50 meters of a swamp (4-fold increase) (Table 2). To a lesser degree, responses to MSP-2 were significantly associated with increasing age and responses to MSP-3 and GLURP-R2 were significantly associated with living near a swamp. As we have previously described decreasing incidence of malaria and increasing risk of treatment failure with AQ+SP over the course of this study [17], [18], we examined associations between calendar time and antibody levels at diagnosis. Surprisingly, none of the measured antibody responses decreased significantly over time to indicate waning immunity. Recent asymptomatic parasitemia, a strong predictor of parasite clearance in previous analyses [12], was positively associated with anti-AMA-1 levels both at Day 0 and Day 14. However, recent asymptomatic parasitemia was also negatively associated with anti-CSP levels at Day 0 and anti-CSP and MSP-1 levels on Day 14. There were minimal associations between parasite density and antibody levels at the time of malaria.

**Table 2:** Associations between epidemiologic measures of *Plasmodium falciparum* exposure and antibody levels.

	Fold Change, Antibody level (95% CI)				
	Age at enrollment(per year)	Living within 50mof swamp	Date sample collected(per year)	Asymptomaticparasitemia withinlast 180 days	Parasite density(per doubling)
CSP D0	1.09 (0.97–1.22)	1.82 (0.95–3.49)	0.86 (0.49–1.50)	**0.48 (0.25–0.91)** *	0.98 (0.86–1.11)
CSP D14	1.05 (0.90–1.21)	1.73 (0.94–3.18)	0.78 (0.47–1.28)	0.27 (0.13–0.55)***	0.99 (0.86–1.13)
LSA D0	1.03 (0.86–1.24)	1.53 (0.61–3.86)	1.38 (0.69–2.77)	1.04 (0.41–2.62)	1.00 (0.84–1.20)
LSA D14	0.97 (0.81–1.17)	1.39 (0.52–3.72)	1.26 (0.56–2.81)	0.60 (0.20–1.81)	1.05 (0.86–1.29)
AMA-1 D0	1.44 (1.20–1.73)***	3.98 (1.56–10.1)**	0.89 (0.47–1.67)	**3.90 (1.23–12.3)** *	0.94 (0.78–1.13)
AMA-1 D14	1.19 (0.97–1.46)	2.07 (0.63–6.68)	1.37 (0.50–3.70)	**5.11 (1.13–23.1)** *	1.03 (0.84–1.27)
MSP-1 D0	1.01 (0.86–1.19)	1.84 (0.78–4.35)	1.37 (0.64–2.94)	0.52 (0.23–1.16)	0.98 (0.84–1.14)
MSP-1 D14	0.98 (0.82–1.18)	0.64 (0.27–1.51)	1.81 (0.87–3.77)	0.27 (0.10–0.68)**	**1.23 (1.01–1.51)** *
MSP-2 D0	**1.22 (1.02–1.46)** *	2.09 (0.93–4.69)	1.45 (0.82–2.56)	1.81 (0.82–4.02)	0.81 (0.68–0.96)**
MSP-2 D14	1.18 (1.00–1.39)	1.06 (0.49–2.28)	1.96 (1.18–3.24)**	1.46 (0.62–3.46)	1.05 (0.88–1.25)
MSP-3 D0	1.08 (0.97–1.21)	**2.17 (1.15–4.08)** *	1.38 (0.90–2.12)	1.85 (0.88–3.92)	0.91 (0.79–1.05)
MSP-3 D14	1.09 (0.96–1.24)	1.26 (0.62–2.56)	1.22 (0.70–2.14)	1.39 (0.61–3.18)	1.08 (0.90–1.28)
GLURP-R0 D0	1.05 (0.94–1.17)	1.65 (0.98–2.78)	1.19 (0.77–1.85)	0.61 (0.31–1.19)	0.90 (0.81–1.00)
GLURP-R0 D14	1.10 (0.99–1.22)	1.23 (0.73–2.09)	1.48 (0.88–2.48)	0.88 (0.43–1.80)	1.03 (0.92–1.16)
GLURP-R2 D0	1.01 (0.87–1.18)	**2.26 (1.07–4.77)** *	1.30 (0.78–2.19)	0.67 (0.32–1.39)	0.95 (0.80–1.12)
GLURP-R2 D14	1.04 (0.88–1.23)	1.50 (0.67–3.32)	1.48 (0.78–2.82)	0.90 (0.38–2.15)	1.09 (0.92–1.30)

Note: weighted multivariate analysis with inference obtained using bootstrapping.

Circumsporozoite protein (CSP); liver stage-antigen (LSA); apical membrane antigen 1 (AMA-1); merozoite surface protein 1, 2, or 3 (MSP-1, MSP-2, MSP-3); amino- or carboxy-terminal region of glutamine rich protein (GLURP-R0, GLURP-R2) levels at the time of malaria diagnosis (D0) or 14 days following malaria diagnosis/treatment (D14).

* p<0.05, **p≤0.01, ***p≤0.001.

### Antibodies Directed Against AMA-1 at the Time of Antimalarial Therapy are Associated with Protection Against Treatment Failure

**Figure 2:**
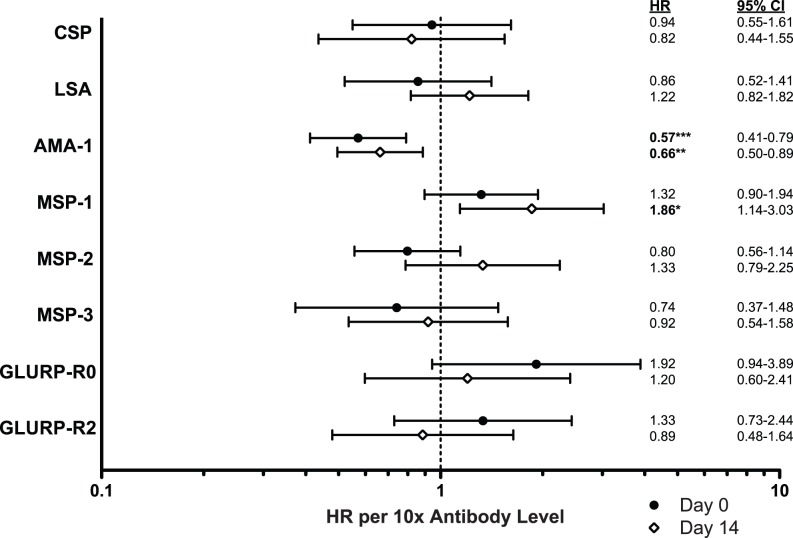
IgG responses and protection against treatment failure with amodiaquine+sulfadoxine-pyrimethamine (AQ+SP). Associations between IgG responses to 8 *Plasmodium falciparum* antigens and protection against treatment failure with AQ+SP were examined. Hazard ratios (HR), depicted by closed circles (Day 0, time of malaria treatment) or open diamonds (Day 14, 14 days following initiation of treatment), are expressed for every 10-fold increase in antibody level. Error bars indicate 95% confidence intervals (CI). Hazard ratios were evaluated using Cox proportional hazards, adjusting for parasite polymorphisms associated with treatment failure and subject age, with robust inference accounting for repeated measures in the same individual. Circumsporozoite protein (CSP); liver stage-antigen (LSA); apical membrane antigen 1 (AMA-1); merozoite surface protein 1, 2, or 3 (MSP-1, MSP-2, MSP-3); amino- or carboxy-terminal region of glutamine rich protein (GLURP-R0, GLURP-R2). *p<0.05, **p≤0.01, ***p≤0.001.

To determine whether antibody responses were associated with the ability to clear parasites after treatment with AQ+SP, we calculated the hazard ratio of treatment failure associated with each response, adjusting for parasite mutations previously shown to be associated with treatment failure. We additionally adjusted for subject age, a strong predictor of treatment failure in these subjects [12] to minimize the chance that antibody responses were merely acting as surrogates of age. Increasing levels of antibodies to AMA-1, but none of the other measured responses, was associated with a significantly lower hazard of treatment failure (Figure 2). Every 10-fold increase in anti-AMA-1 levels at Day 0 was associated with an additional 43% protection against treatment failure (HR 0.57, CI: 0.41–0.79, p = 0.001). Significant associations were also seen if AMA-1 was assessed as a dichotomous variable (HR 0.36 for positive vs. negative, CI: 0.16–0.82, p = 0.015) or in tertiles (HR 0.19 for highest vs. lowest tertile, CI: 0.06–0.63, p = 0.007). Similar protection was associated with anti-AMA-1 levels measured on Day 14 (HR 0.66 per 10-fold increase, CI: 0.50–0.89, p = 0.006). Surprisingly, increasing levels of MSP-1 on Day 14 were associated with an increased risk of treatment failure (HR 1.86, CI: 1.14–3.03, p = 0.013). The degree of boosting of antibody levels from Day 0 to Day 14 was not associated with protection for any of the responses measured (data not shown). The number of antigens to which an individual responded on Day 0 was not significantly associated with protection when evaluated as a continuous variable (HR 0.89 per additional response, p = 0.3) and weakly associated when evaluated as a dichotomous variable, though less so than the dichotomous response to AMA-1 alone (HR 0.46 for 6–8 vs. 5 or fewer responses, CI: 0.18–1.23, p = 0.12). The competing risk of new infection, which occurred by day 63 in 22% of cases, was not likely to bias the association between antibodies to AMA-1 and the risk of treatment failure via informative censoring (HR for new infection 1.0 per 10-fold increase, p = 0.9).

**Figure 3:**
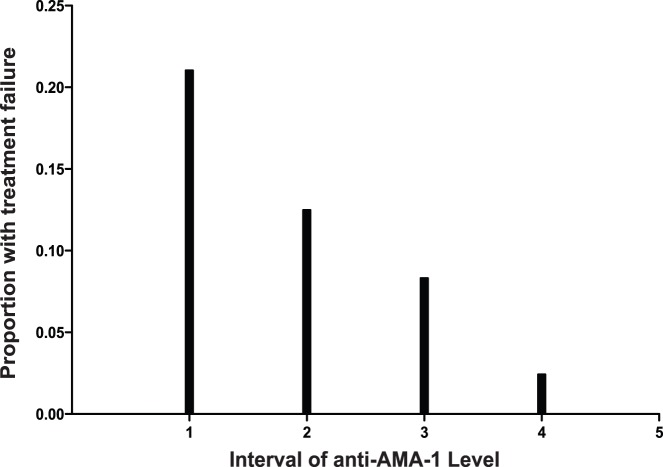
Relationship between anti-apical membrane antigen 1 (AMA-1) antibody levels and risk of treatment failure. A consistent relationship was seen across the range of values observed. Anti-AMA-1 levels (log-transformed) were divided into five equal intervals, each representing a 10-fold increase in level.

To further examine the dose-response relationship between anti-AMA-1 levels on Day 0 and treatment failure, antibody levels were divided into 5 equal intervals, each representing a 10-fold increase. There was a consistent, fairly linear relationship between anti-AMA-1 levels and protection against treatment failure over the entire range of measured responses (Figure 3). All malaria treatments in children with responses in the top 36% of the range were associated with successful clearance of parasites.

Our longitudinal study design allowed us to analyze the effect of changes in antibody responses to AMA-1 measured during different malaria episodes in the same subject, with 200 out of 244 episodes occurring in subjects with more than one episode. In these 200 malaria episodes, antibody responses to AMA-1 measured at the time of malaria diagnosis were highly correlated within individuals (intraclass correlation 0.88, CI: 0.82–0.92) and showed no consistent change within individuals over calendar time (fold change 1.18 per year, CI 0.8–1.75, p = 0.4). Interestingly, anti-AMA-1 levels measured at the first episode of malaria in each subject were similarly predictive of treatment failure for all subsequent episodes in that subject (HR 0.37 per 10-fold increase, CI: 0.19–0.73, p = 0.004) as anti-AMA-1 values measured at the time of the subsequent episodes themselves (HR 0.44, CI: 0.25–0.78, p = 0.001). Multivariate analysis of these 137 subsequent episodes indicated that the change in anti-AMA-1 level from the initial episode to the subsequent episode provided no additional information once the initial measurement was included (p = 0.9).

## Discussion

We measured IgG responses to 8 *P. falciparum* antigens at the time of treatment of malaria with AQ+SP to determine whether any of these responses were associated with protection from treatment failure. We found that higher antibody responses to AMA-1, but to none of the other 7 antigens tested, were significantly associated with protection. Furthermore, we found a strong dose response relationship, with higher responses to AMA-1 consistently associated with increased protection against treatment failure.

Does the identified strong association between AMA-1 antibody level and clearance of parasites suggest a causal role in protection? Antibody levels against AMA-1 have been associated with protection from clinical malaria in this [9] and other cohorts [6], [15]. In addition, data from a recent malaria vaccine trial suggest that vaccination with FMP2.1/AS02_A_, a recombinant protein based on AMA-1, may induce strain-specific protection [30]. However, an alternative, though not mutually exclusive, explanation of our findings is that antibodies against AMA-1 provide information as a biomarker of immunity beyond any causal effect. Indeed, we found strong associations between epidemiological estimates of *P. falciparum* exposure and anti-AMA-1 levels at the time of symptomatic malaria. Furthermore, antibody responses to all 8 antigens were significantly correlated with each other. In addition, despite our prior observation of decreasing efficacy of AQ+SP over time that was likely due to waning immunity [12], antibody responses to AMA-1 at the time of malarial illness did not decrease significantly over time. Furthermore, analysis of anti-AMA-1 levels across multiple episodes of malaria in the same subject suggested that variation within a subject had no association with treatment outcomes. These data argue against a causal effect, but suggest a more robust predictive value of anti-AMA-1 as a biomarker. Thus, antibody responses to AMA-1 may assist in clearance of parasites, but our data more strongly suggest that they offer a useful individual-level biomarker of blood stage immunity.

Associations between antibody responses to various antigens and antimalarial treatment outcome have been examined in prior studies [31]–[39]. These studies have found associations between positive or high IgG responses to a number of antigens (NANP repeat of CSP [39], K1 and MAD20 block 2 variants of MSP-1 [34], MSP-1_19_ [32], [33], and GLURP-R0 and R2 [36]) and protection from treatment failure. Three of these studies included assessment of AMA-1 antibodies in their analyses, but these did not show strong associations between anti-AMA-1 levels and clearance of parasitemia [33], [36], [37]. Differences between our results and prior studies may be attributable to the antimalarial therapy used, due to varying efficacy and pharmacokinetics; differences in the epidemiological setting and patient characteristics; different means of assessing treatment outcome; and the use of different laboratory and analytical methods. It should also be noted that while response to antimalarial therapy may provide information on blood stage protection not directly confounded by prior exposure, such an analysis is inherently limited to subjects who are exposed enough to *P. falciparum* to observe a malaria episode and do not have complete immunity against symptomatic malaria. Of note, we obtained highly quantitative values for each response and demonstrated strong associations between anti-AMA-1 levels and protection, whether analyzing responses as reactive/non-reactive, in tertiles, or as a continuous variable.

Interestingly, we found that, for anti-MSP-1 levels assessed 14 days following malaria treatment, lower levels were associated with clearance of parasites. This finding is in contrast to previous studies that found anti-MSP-1_19_ levels at the time of treatment to be protective against treatment failure [32], [33]. While our finding may represent a spurious result, given the marginal significance when taking into account multiple testing, there are some biologically plausible mechanisms for this seemingly paradoxical association. For example, persistent parasite replication in the blood after antimalarial therapy may have stimulated responses to MSP-1 on Day 14. Supporting this hypothesis, anti-MSP-1 levels were higher and boosted more from Day 0 to Day 14 in those children with higher parasite densities at diagnosis. Another possibility is the presence of blocking or neutral antibodies that compete for binding sites on the C-terminal epidermal growth factor domain of MSP-1_19_ [40]. Binding of these antibodies (i.e. prevention of beneficial “inhibitory” antibodies that inhibit invasion and secondary processing of MSP-1) lead to MSP-1_19_ processing and resultant invasion of the red blood cell. Indeed, a study in Nigerian children revealed that there was evidence for both blocking and neutral antibodies competing for MSP-1_19_ binding sites as there was absent inhibitory activity observed despite high MSP-1_19_ prevalence and levels by ELISA [41].

In conclusion, we have shown that the levels of antibodies directed against AMA-1 at the time of presentation with malaria are strongly associated with concurrent and future blood stage immunity, as measured by the ability of the host to clear *P. falciparum*. A mechanistic role in protection is possible, but would need to be evaluated in the context of broader assessment of immunologic responses. The strong dose-response relationship we observed indicates that quantitative measurement of these antibodies may offer a robust biomarker of immunity and should be validated in other epidemiologic settings.
